# Nano Geochemistry

**DOI:** 10.3390/nano12071039

**Published:** 2022-03-22

**Authors:** Thorsten Schäfer, Woojin Lee, Gopala Krishna Darbha

**Affiliations:** 1 Friedrich Schiller University Jena, Institute of Geosciences, Applied Geology, 07749 Jena, Germany; 2 Civil and Environmental Engineering, Nazarbayev University, Nur-Sultan 010000, Kazakhstan; woojin.lee@nu.edu.kz; 3 Indian Institute of Science Education and Research Kolkata, Mohanpur, West Bengal 741246, India; gkdarbha@iiserkol.ac.in

It is our great pleasure to briefly introduce our motivation to collect scientific contributions for this Special Issue, entitled “Nano Geochemistry”. The geophysical and chemical dynamics at the solid–water interface, ultimately, control the transport properties of natural and engineered colloids/nanoparticles via, e.g., mineral dissolution/precipitation reactions and the variation in nano- to microscale surface roughness [1,2]. The nanoparticles present can significantly influence the mobility of strongly sorbing organic and/or inorganic contaminants in groundwater systems, frequently used as a drinking-water resource [3]. In addition, wetting/drying cycles in the vadose zone have attracted interest, concerning the mobility of organic nanoparticles/colloids [4], and the nanoparticle composition and element redox state can significantly change, especially in karst systems, due to seasonal variations [5]. More pronounced changes, due to extreme weather events, potentially triggered by climate change, have been observed [6]. The generation of these nanoparticles based on the nucleation and growth theory (classical or non-classical crystallization pathway) for the formation of nanoparticles in natural systems is still a matter of debate. It could be shown, e.g., for magnetite, that the nanoparticle formation in natural systems proceeds through rapid agglomeration of nanometric primary particles. In contrast to the nucleation of other minerals, no intermediate bulk phase is involved [7]. Nucleation and nanoparticle formation, associated with surfaces, are also key aspects of the formation of, e.g., Au ore deposits and hydrothermal vents [2,8], also referred to as the field of nanogeology [8].

Biomineralization is another field of active research, especially the study of magnetotactic bacteria (MTB), which attracts great interest. Due to their nano-sized magnetosomes (MS), which are biomineral crystals of either magnetite (Fe_3_O_4_) or greigite (Fe_3_S_4_), with a size range of 30–120 nm, the removal of heavy metals from wastewater, via an external magnetic field, might be very promising [9]. However, detailed investigations on, e.g., the economic suitability and cost-effective magnetic separator for metal recovery, amongst other things, are needed.

The shortage of freshwater, due to surface and groundwater contamination, is still creating a need for novel and sustainable water purification technologies in the global environmental technology market [10]. Diverse environmental technologies, such as adsorption and ion exchange, reverse osmosis, electro-catalysis, and biological redox processes, have all been developed, showing disadvantages to date. Thus, the market and environmental engineers have been looking for effective, promising water purification technologies, to satisfy novel operational, technological, and economic needs, with higher removal efficiency and selectivity. Nanomaterials bring a new hope towards efficient water purification, due to their high surface area and reactivity. In addition, advanced remediation strategies, using nano zero-valent iron (nZVI) or modified core-shell type functionalized nanoparticles, are available to decontaminate groundwater resources, under the prevailing geochemical conditions of the natural systems [11,12].

Extensive geochemical knowledge on the speciation of targeted contaminants in the environment can aid in the further fine-tuning of nanoadsorbents, leading to high selectivity and removal efficiency [13].

Among various nanoadsorbents, redox-sensitive nanoparticles (RSNPs) and composites can feature electron transfer on their surface, resulting in the redox transformation of contaminants. Direct electron gain by heavy metal ions and other contaminants can result in their reductive co-precipitation, while free radical generation in aqueous solutions can result in the degradation of organic contaminants [14,15]. nZVI and its composites are among the extensively explored RSNPs, for the remediation of contaminated soil and groundwater, and have brought about the revolution of remediation technologies [16], leading to the breakthrough of advanced water purification technologies, with diverse nanomaterials and their redox catalysis [17]. Extensive studies on the nanomaterial applications and natural and anthropogenic nano-catalysis have been carried out to remove heavy metals [18], organic chemicals [19,20], and radionuclides [21], effectively and selectively in the contaminated soil and groundwater plumes.

Direct application of these RSNPs is challenging due to their instantaneous oxidation; therefore, researchers are continuously exploring various methods for the preservation of RSNPs, such as encapsulation in polymers, use of supporting surfaces, like clays and carbon-based materials, antioxidant capping, etc., to prevent agglomeration and preserve their redox activity [11,12,22,23]. Moreover, anthropogenically influenced soils and water bodies may hold multiple contaminant loads. Therefore, the simultaneous removal or stabilization of multiple pollutants, with advanced redox-sensitive nanocomposites, is at the forefront of current scientific focus [14,23].

In industrial processes, e.g., the global cement industry, different raw materials, called supplementary cementitious materials (SCMs), are considered to replace part of the clinker in cement, which is discussed as one of the most successful strategies to reduce CO_2_ emissions globally [24]. Clays (montmorillonite, illite, kaolinite), nanoscale in nature, are the only material available in the quantities needed to meet the SCM demand, especially in those countries where a growth in demand for cement is forecast [24]. However, early cement hydration and rheology are key aspects [25,26], and future research directions need the mechanistic understanding of the SGM reaction pathways and the new design of appropriate superplasticizers. The mechanistic process understanding of nanomaterials is a prerequisite for the reliable prediction of the long-term behavior of the chemical compounds in the natural and engineered environment. Due to the advancement of high-energy X-ray beam spectroscopy (XAF and XANES) and supercomputing systems with molecular dynamic and DFT simulations, the reaction mechanisms of environmental contaminants on the surface of natural and anthropogenic nanomaterials could be more conveniently observed and identified, and the prediction of their molecular behaviors would provide more credible information on the nanomaterial applications for the successful remediation of contaminated underground environments [18,27].

## Data Availability

Not applicable.
